# The Independent Associations of Professional Autonomy, the Work Environment, and Role Ambiguity with Job Satisfaction Among Korean Nurses in Physician-Support Roles: A Cross-Sectional Study

**DOI:** 10.3390/healthcare14183037

**Published:** 2026-09-16

**Authors:** Ju Young Kim, Yoon Jeong Choi

**Affiliations:** 1 Division of Nursing, Inje University Busan Paik Hospital, Busan 47392, Republic of Korea; 2 College of Nursing, Inje University, Busan 47392, Republic of Korea

**Keywords:** physician assistants, nursing staff, hospital, nurse’s role, scope of practice, professional autonomy, working conditions, role ambiguity, job satisfaction

## Abstract

**Background/Objectives:** Korean hospitals have deployed nurses in physician-support roles since 1997, and more so since the physicians’ collective action in 2024, yet the role lacked a clear legal definition until the Nursing Act and its enforcement rule provided one. This study asked which of professional autonomy, the work environment, and role ambiguity remains independently associated with job satisfaction when the three are considered together. **Methods:** A total of 130 nurses in these roles at two Korean university hospitals completed a cross-sectional survey in July–August 2025, before that rule took effect, using Korean versions of the Dempster Practice Behaviors Scale, Nurse Practitioner Primary Care Organizational Climate Questionnaire, Role Ambiguity Scale, and Job Satisfaction Scale for Clinical Nurses. **Results:** Means were 3.40/5 (professional autonomy), 2.61/4 (work environment), 2.98/5 (role ambiguity), and 3.56/5 (job satisfaction), and the first three correlated with job satisfaction (*r* = 0.67, 0.67, and −0.51; all *p* < 0.001). Adjusted for six covariates, professional autonomy (*β* = 0.42) and the work environment (*β* = 0.37; both *p* < 0.001) remained independently associated with job satisfaction, explaining 55.6% of the variance, whereas role ambiguity did not (*β* = −0.11, *p* = 0.135). **Conclusions:** Professional autonomy and the work environment were the constructs independently associated with job satisfaction once the three were considered together. Attention should therefore focus on the institutional conditions that widen these nurses’ discretion over their own work and on their relationships with administrators, the lowest-scoring domain of the work environment. Longitudinal research is required to establish whether these associations are causal.

## 1. Introduction

Korean healthcare institutions have deployed registered nurses in physician-support roles since 1997, in response to the reluctance of medical graduates to enter major clinical specialties, the resulting imbalance in the supply of resident physicians, and rising patient demand for medical services [1]. Awareness of the need for this workforce grew further when the enactment of the Nursing Act coincided with the departure of resident physicians and collective action by physicians in February 2024, which created substantial gaps in service delivery [2]. The Nursing Act took effect on 21 June 2025 [3], and the enforcement rule on nurse-delivered physician-support work, promulgated on 10 July 2026 and in force from 11 August 2026, subsequently specified the eligibility, scope of practice, and institutional governance of the nurses who carry out this work [4]. For roughly three decades before that rule, the role existed without a statutory basis.

In the United States, the physician assistant is a distinct licensed health profession: practitioners complete an accredited graduate program, pass a national certifying examination, obtain state licensure, and deliver patient care within a legally defined scope of practice in collaboration with physicians [5]. In Korea, by contrast, nurses in physician-support roles were introduced through the discretionary arrangements of individual institutions, without a clear legal definition or institutional basis, and this has generated persistent uncertainty about the boundaries of their work and about where accountability lies [1,6]. The difference is also one of nomenclature. These nurses have conventionally been called “physician assistant nurses” or “PA nurses” in Korea, and that designation appears in much of the Korean literature cited here, but they are registered nurses rather than members of the licensed physician assistant profession, and the International Council of Nurses has cautioned against using the two designations interchangeably [7]. The title is unsettled in the United States as well: after the American Academy of Physician Assistants, now the American Academy of Physician Associates, voted in May 2021 to change the title of the profession [8], the certifying body has continued to write “physician assistants/associates” and to retain “assistant” in its corporate name and in the PA-C (Physician Assistant-Certified) credential [5]. The enforcement rule names them *jinryo-jiwon jeondam ganhosa*, nurses dedicated to physician-support work [4]. This article therefore uses the descriptive term “nurses in physician-support roles” throughout and reserves “physician assistant” for the licensed profession as constituted outside Korea.

Job satisfaction among nurses refers to an individual’s subjective level of satisfaction with their organization and their work, and is a core determinant of the efficiency of nursing organizations and the quality of health services [9]. Previous research has linked job satisfaction among nurses in physician-support roles to the absence of a legal basis and of clear work regulations [10]. Nurses in physician-support roles work without clearly defined role boundaries and are therefore exposed to role conflict, which has been discussed as weakening professional identity [11]. Role ambiguity has likewise been reported as an antecedent of lower job satisfaction [12]. Job satisfaction in this group has also been reported to vary with a range of work-related factors, including job stress [13,14], role stress and role conflict [15], and the work environment [16]. Of the work-related factors examined in this literature, the present study addresses professional autonomy, the work environment, and role ambiguity, each of which has recurred in the literature as an independent correlate in its own right. Professional autonomy is the degree of control a nurse has over their own work, the work environment is the organizational setting that surrounds it, and role ambiguity is how clearly the content of the work and the standards by which it is judged are defined [12]. Autonomy has been regarded as a key step toward full professional status in nursing [17], yet among nurses in physician-support roles in Korea it has correlated with job satisfaction while failing to retain an independent association once other work-related variables were entered into the model [18,19]. The work environment extends to organizational policy, collegial relations, and institutional support [20]; favorable environments have been associated with lower turnover intention [21], and in nurses in physician-support roles the work environment has been associated with job satisfaction [16]. Role ambiguity has been linked to role conflict, job stress and lower job satisfaction [22,23]; nurses in physician-support roles are unusually exposed to it, because limited organizational clarity about the role invites conflict with other professions [24].

**Figure 1:**
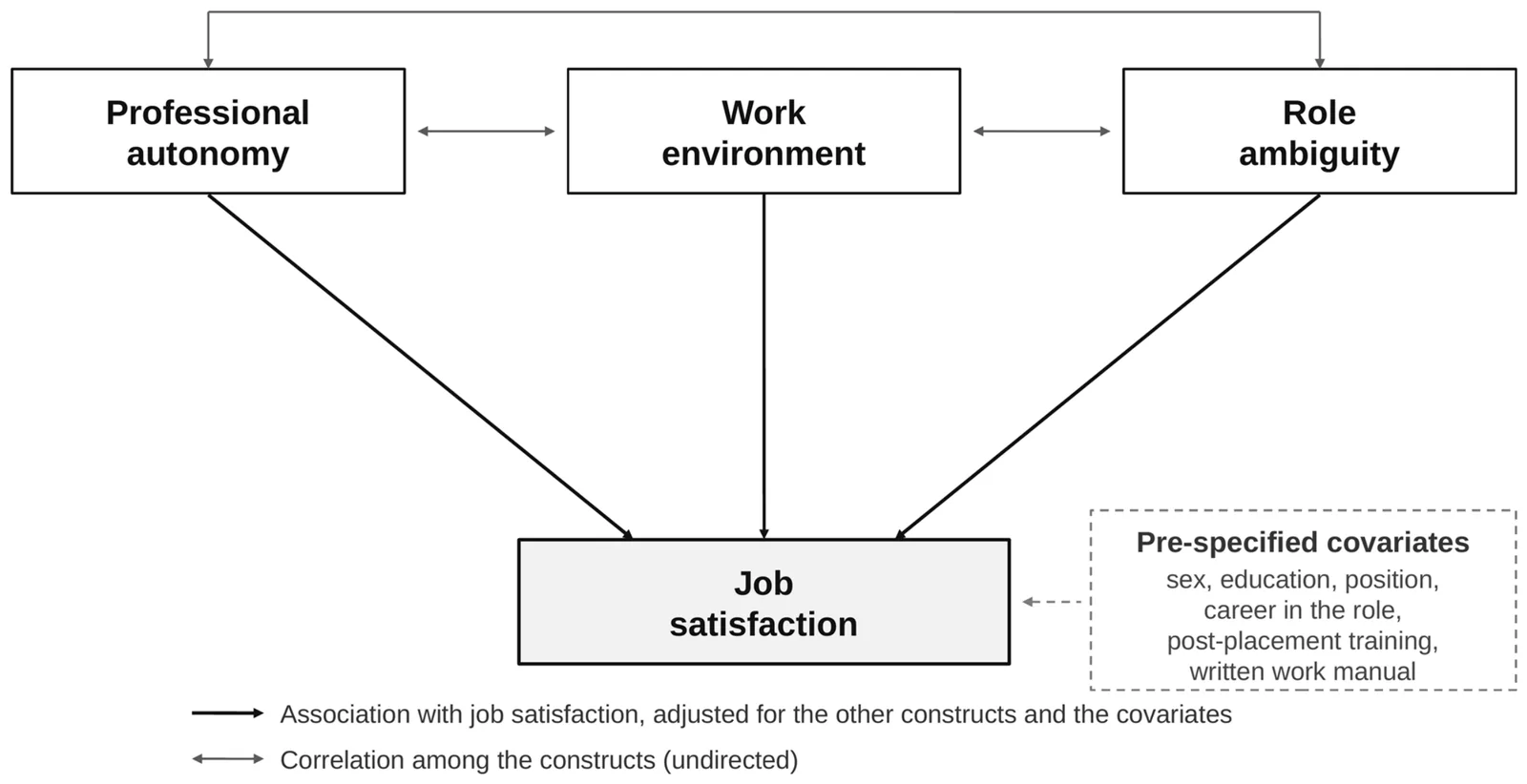
A conceptual model of the analysis. The links among professional autonomy, the work environment, and role ambiguity are shown as undirected because the design does not identify their temporal ordering, and the arrows denote statistical association rather than causal effect.

The three are unlikely, however, to be wholly independent of one another. The work environment has been argued to bound the autonomy that can actually be exercised [25], and in role theory role ambiguity is held to originate in what an organization conveys about a position [26]. The analysis reported here treats the three constructs as interrelated without assuming any ordering among them (Figure 1). In a role established by institutional discretion rather than by statute, where no defined scope is available and jurisdiction between occupations is settled by informal negotiation rather than by rule [27], the three are likely to appear together. Korean research has nonetheless consisted largely of surveys of operational status or of studies examining one or two of these factors at a time [1,16]. Apart from the two studies noted above, professional autonomy has been assessed only at the level of correlation [28]; the work environment has been examined without reference to autonomy [29]; and role ambiguity has been studied mainly among general ward nurses [23]. With the boundaries of the role indistinct [30], it is not known which of the three carries independent weight for job satisfaction once the others are taken into account.

This study examined professional autonomy, the work environment, and role ambiguity among Korean nurses in physician-support roles, and asked which of them remains independently associated with job satisfaction once the others and a set of pre-specified characteristics are taken into account. Data were collected after the Nursing Act had taken effect but before the enforcement rule specifying scope of practice did. The findings therefore describe this workforce in the final months of the arrangement that governed it for nearly three decades, and provide a reference point against which the effects of the new framework can later be assessed. The originality of this study lies in estimating the mutually adjusted associations of these three constructs in a workforce observed at a defined moment of regulatory transition. The sequence in which a health system facing a shortage of physicians shifts tasks to nurses and only later builds the legal framework that defines the resulting role is not specific to Korea, so the question of which conditions sustain job satisfaction in such a role arises in any system where task-shifting outpaces regulation. The estimates reported here are nonetheless specific to this institutional and national context and to this point in time, and replication in other settings is required before they are treated as general.

## 2. Materials and Methods

### 2.1. Study Design

This was a cross-sectional descriptive study designed to describe levels of professional autonomy, work environment, role ambiguity, and job satisfaction among nurses in physician-support roles, and to examine the associations of these variables with job satisfaction. Reporting follows the Strengthening the Reporting of Observational Studies in Epidemiology (STROBE) statement for cross-sectional studies [31].

### 2.2. Setting and Participants

Participants were nurses in physician-support roles at two university hospitals in a metropolitan city in southeastern Korea with a population of approximately 3.3 million. Five university hospitals operate in that city, ranging in size from approximately 840 to 1200 beds; three of these, including both participating hospitals, have approximately 900 beds. The two participating hospitals were selected on the basis of institutional access, which allowed their nursing department to be approached directly. Both agreed to participate and no other institution was approached. The sample was therefore one of convenience and is not representative of the national workforce in this role. Eligible participants were currently engaged in physician-support work and had at least six months of experience in the role. Nurses whose duties were not directly related to physician-support work, namely quality improvement administrative nurses, infection control administrative nurses, wound care nurses, and organ transplantation coordinators, were excluded. All participants received an explanation of the purpose and methods of the study and volunteered to take part.

### 2.3. Sample Size

The required sample size was calculated using G*Power version 3.1.9.7 (Heinrich Heine University Düsseldorf, Düsseldorf, Germany) for multiple regression analysis. With a significance level of 0.05, power of 0.80, a medium effect size of 0.15, and nine predictors (six general characteristics—sex, educational attainment, position, career in the physician-support role, receipt of job training after placement, and the availability of a written work manual—together with professional autonomy, work environment, and role ambiguity), the minimum required sample was 114. This calculation was performed for the overall regression model and not for the unique contribution of any individual predictor. Allowing for 20% incomplete responses, questionnaires were distributed to 143 nurses. After excluding 13 questionnaires that were incomplete because of missing items, 130 were analyzed.

In July 2025, the two hospitals employed 177 and 179 nurses in physician-support roles, 356 in total. A total of 100 questionnaires were provided at the first hospital and 43 at the second, 143 in total, or 40.2% of those employed, reflecting the number reachable through each department of nursing during the recruitment period. All 143 were returned, 13 were excluded because of missing items, leaving 130 for analysis, or 90.9% of those provided and 36.5% of those employed. The proportion provided with a questionnaire differed between the two sites, 56.5% and 24.0%, so the analytic sample is likely to over-represent the site with the higher proportion.

### 2.4. Measures

#### 2.4.1. General Characteristics

General characteristics were collected using an investigator-developed questionnaire covering age, sex, highest educational attainment, marital status, whether the participant had children, position, total clinical career, career in the physician-support role, clinical department, organizational affiliation, working type, motivation for entering the role, annual salary, receipt and duration of job training before and after placement in the role, and the availability of a written work manual. Total clinical career and career in the physician-support role were reported in years and months and were rounded to whole years for analysis. The questionnaire offered “emergency department” and “other” as response options for clinical department, but no participant selected either.

#### 2.4.2. Professional Autonomy

Professional autonomy was measured with the Dempster Practice Behaviors Scale (DPBS), developed by Dempster [32] for advanced practice nurses and translated into Korean by Kim [33]. The instrument comprises 30 items across four subscales: readiness (11 items), empowerment (7 items), actualization (9 items), and valuation (3 items). Items are rated on a 5-point Likert scale from 1 (strongly disagree) to 5 (strongly agree), with higher scores indicating a stronger perception of professional autonomy. Items 8, 13, 17, and 28, all belonging to the empowerment subscale, were reverse-scored before analysis. Cronbach’s alpha was 0.92 in the study by Kim [33] and 0.95 in the present study.

#### 2.4.3. Work Environment

The work environment was measured with the Nurse Practitioner Primary Care Organizational Climate Questionnaire (NP-PCOCQ), developed by Poghosyan et al. [34] for nurse practitioners and adapted for Korean nurses in physician-support roles by Ryu et al. [29]. The instrument was originally developed to measure the organizational climate perceived by nurse practitioners and was named a work environment scale in the Korean adaptation [29]. It comprises 29 items across four subscales: professional visibility (4 items), relationships with administrators (9 items), relationships with physicians (7 items), and independent practice and support (9 items). Items are rated on a 4-point Likert scale from 1 (strongly disagree) to 4 (strongly agree), with higher scores indicating a more favorable perception of the work environment. Cronbach’s alpha was 0.93 in the study by Ryu et al. [29] and 0.94 in the present study.

#### 2.4.4. Role Ambiguity

Role ambiguity was measured with the Role Ambiguity Scale, developed by Rizzo et al. [12] for organizational members and translated into Korean by Choi et al. [35]. The instrument comprises seven items rated on a 5-point Likert scale from 1 (strongly disagree) to 5 (strongly agree), with higher scores indicating greater role ambiguity. The items of the original scale are worded in the direction of role clarity and therefore require reverse scoring to index ambiguity. The Korean seven-item version used here is worded in the direction of ambiguity, so that agreement with an item indicates that the content or standards of the work are less clearly defined; accordingly, no item was reverse-scored, and the scale score is the unweighted mean of the seven raw item scores. An item-level audit of the raw data confirmed this direction: every item correlated positively with the mean of the remaining items (corrected item–total *r* = 0.42 to 0.82). Cronbach’s alpha was 0.88 in the study by Choi et al. [35] and 0.89 in the present study.

#### 2.4.5. Job Satisfaction

Job satisfaction was measured with the Job Satisfaction Scale for Clinical Nurses developed by Lee et al. [9]. The instrument comprises 33 items across six subscales: organizational recognition and professional achievement (9 items), personal maturity through work (6 items), interpersonal relationships based on respect and recognition (8 items), fulfillment of responsibility as a professional nurse (4 items), exercise of professional competence (3 items), and job security and reward (3 items). Items are rated on a 5-point Likert scale from 1 (strongly disagree) to 5 (strongly agree), with higher scores indicating greater job satisfaction. Cronbach’s alpha was 0.95 in the study by Lee et al. [9] and 0.95 in the present study.

### 2.5. Data Collection

Data were collected between 11 July and 8 August 2025, following approval by the Institutional Review Board. The researchers explained the purpose, methods, and procedures of the study to the department of nursing at each hospital and obtained its cooperation. One hundred questionnaires were provided to the department of nursing at one hospital and 43 at the other. Nurses in physician-support roles are not organized as a single nursing unit but work across clinical departments, and the department of nursing placed the questionnaires in the departments in which they work so that those who understood the purpose of the study and wished to take part could take one themselves. No manager selected who received a questionnaire or was in a position to know who had taken one, and no manager took part in the response process at any stage. Respondents were informed in writing that participation was voluntary, that declining carried no disadvantage. All participants signed a written consent form before completing the questionnaire; consent forms were returned inside the same envelope as the questionnaire, and the researchers separated the two immediately on opening and stored the consent forms separately from the questionnaires. Completed questionnaires carried no identifying information and were placed in sealable envelopes to protect personal information. Each respondent was given an individual envelope and sealed it personally. On a set date the department of nursing at each hospital collected the sealed envelopes together, and the researchers collected them from the department of nursing in person. The envelopes remained sealed until they reached the researchers, so no manager and no member of the department of nursing saw any individual response. The questionnaire did not record the hospital at which each respondent worked. Several of the general characteristics collected, among them clinical department, department of affiliation, position, and educational attainment, fall in categories containing fewer than ten respondents, and a two-level site indicator would subdivide those categories to the point at which an individual respondent could be identified within their own institution, together with their job satisfaction and their ratings of relationships with administrators and physicians. Site was therefore not recorded, and for the same reason participant numbers and study variables are not reported separately by hospital.

### 2.6. Data Analysis

Data were analyzed using IBM SPSS Statistics for Windows version 29.0 (IBM Corp., Armonk, NY, USA), except for the power calculations, which used G*Power 3.1.9.7. General characteristics were summarized as frequencies and percentages or as means and standard deviations. Study variables were summarized as means, standard deviations, minimums, and maximums. Differences in job satisfaction by general characteristics were tested with independent-samples *t*-tests and one-way analysis of variance with Scheffé post hoc tests. Correlations were examined with Pearson coefficients, and variables associated with job satisfaction with multiple linear regression using the enter method. A two-sided *p*-value below 0.05 was considered statistically significant. Thirteen questionnaires with missing items were excluded before analysis, and the 130 retained cases were complete on every variable, so all analyses used the full sample. Variance explained is reported throughout as the adjusted R^2^.

Covariates were specified in advance on conceptual grounds rather than by bivariate screening. Variables were eligible if they preceded the perceptions under study, were plausibly related to both those perceptions and job satisfaction, and did not lie on the causal pathway between them; on this basis sex, educational attainment, position, career in the physician-support role, receipt of job training after placement, and the availability of a written work manual were entered alongside professional autonomy, work environment, and role ambiguity. Because the three main variables may be interrelated, so that their contributions overlap, two supplementary procedures were carried out. The unique contribution of each was quantified as the reduction in *R*^2^ on its removal from the full model. Each main variable was also entered alone and then with the other two added in turn, so that any change in its coefficient could be traced. The first procedure was computed within the full model and therefore retained the covariates; the second did not include them, since its purpose was to trace the overlap among the three variables under study, and its estimates are accordingly not comparable with the covariate-adjusted coefficients of the main regression model. Because professional autonomy, the work environment, and role ambiguity are theoretically interrelated (Figure 1) and were measured at the same time point, each coefficient is a conditional rather than a total association: adjusting for one construct removes any part of another’s association with job satisfaction that runs through it. The analysis therefore identifies which constructs carry an independent association once the others are held constant, but cannot establish which is causally prior.

Regression assumptions were examined beyond the collinearity and independence checks reported below. Normality of residuals was assessed with the Shapiro–Wilk test and with skewness and kurtosis; homoscedasticity with the Breusch–Pagan test; functional form with the Ramsey RESET test; and influence with Cook’s distance, leverage values, and externally studentized residuals. As sensitivity analyses, the primary model was re-estimated with heteroscedasticity-consistent (HC3) standard errors and with percentile bootstrap confidence intervals from 5000 resamples. Because the hospital of recruitment was not recorded, its potential influence was examined through a quantitative bias analysis in which a hypothetical site indicator was constructed 5000 times and entered into the primary model as an additional predictor; the construction is set out in full in Appendix A.

Common method bias was addressed procedurally as far as a single-survey design allowed: the questionnaire was anonymous and self-administered, collected no identifying information, and was returned under the safeguards described in Section 2.5, and the four instruments were administered in their published response formats, which differ, so no single uniform scale ran through the questionnaire. It was then examined post hoc with Harman’s single-factor test, in which all items from the four instruments were entered into a single unrotated exploratory factor analysis.

### 2.7. Ethical Considerations

The study was approved by the Institutional Review Board of Inje University (approval number 2025-04-040-003) and was conducted in accordance with the Declaration of Helsinki. Participants were informed of the purpose of the study and of the guarantee of anonymity and confidentiality, and were told that they could withdraw at any time while completing the questionnaire without any disadvantage. Participant information was stored securely in encrypted files in accordance with the Personal Information Protection Act. Collected data were encrypted and managed so that only the principal investigator could access them, and will be retained for three years after the end of the study and then permanently deleted.

## 3. Results

### 3.1. General Characteristics of Participants

The mean age of participants was 32.73 years, and the largest group was aged 30 years or younger (39.2%). Women accounted for 73.1% of participants and 93.8% held a bachelor’s degree or lower as their highest qualification. Half were unmarried (50.8%) and a third had children (33.1%). Most held the position of staff or charge nurse (78.5%), worked fixed daytime hours (91.5%), and were organizationally affiliated with the department of nursing (97.7%); surgical departments accounted for 63.8%. Mean total clinical career was 9.75 years and mean career in the physician-support role was 5.28 years. Asked why they had entered the role, 38.5% gave professional reasons, an equal proportion cited avoiding rotating shifts, and 23.1% had been assigned by the hospital or gave another reason. Pre-placement job training had been received by 10.8%, who reported a mean of 10.29 h, and post-placement training by 49.2%, who reported a mean of 8.72 h. A written work manual was available to 63.8% (Table 1).

### 3.2. Levels of the Study Variables

The mean score for professional autonomy was 3.40 (standard deviation [SD] 0.65) out of 5, with subscale means in the order of actualization (3.71), valuation (3.43), readiness (3.35), and empowerment (3.05). The mean score for the work environment was 2.61 (SD 0.49) out of 4, with subscale means in the order of relationships with physicians (2.94), independent practice and support (2.74), professional visibility (2.62), and relationships with administrators (2.22). The mean score for role ambiguity was 2.98 (SD 0.87) out of 5. The mean score for job satisfaction was 3.56 (SD 0.56) out of 5, with subscale means in the order of job security and reward (3.95), fulfillment of responsibility (3.92), interpersonal relationships (3.68), exercise of professional competence (3.59), personal maturity (3.54), and organizational recognition and achievement (3.17) (Table 2).

### 3.3. Differences in Job Satisfaction According to General Characteristics

Job satisfaction differed significantly only by receipt of job training after placement in the physician-support role (*t* = 2.13, *p* = 0.035); participants who had received such training reported higher job satisfaction than those who had not. Job satisfaction also tended to decline across the three categories of motivation for entering the role, from 3.69 among those citing professional reasons to 3.50 among those seeking to avoid rotating shifts and 3.43 among those assigned by the hospital, although the difference did not reach significance (*F* = 2.49, *p* = 0.087). No other characteristic was associated with job satisfaction (Table 3).

### 3.4. Correlations Among the Study Variables

Job satisfaction was positively correlated with professional autonomy (*r* = 0.67, *p* < 0.001) and with the work environment (*r* = 0.67, *p* < 0.001), and negatively correlated with role ambiguity (*r* = −0.51, *p* < 0.001). Role ambiguity was negatively correlated with professional autonomy (*r* = −0.48, *p* < 0.001) and with the work environment (*r* = −0.59, *p* < 0.001), and professional autonomy was positively correlated with the work environment (*r* = 0.60, *p* < 0.001) (Table 4).

### 3.5. Variables Associated with Job Satisfaction

Nine variables were entered simultaneously: professional autonomy, work environment, and role ambiguity, together with the six pre-specified covariates, with categorical covariates coded as dummy variables. Independence of residuals and collinearity were within conventional limits: the Durbin–Watson statistic was 2.11, tolerance ranged from 0.50 to 0.98, and variance inflation factors from 1.02 to 1.99; the remaining regression assumptions are reported below.

Professional autonomy (*β* = 0.42, *p* < 0.001), the work environment (*β* = 0.37, *p* < 0.001), and career in the physician-support role (*β* = −0.15, *p* = 0.024) were independently associated with job satisfaction. The associations of professional autonomy and the work environment were positive, whereas job satisfaction was lower among participants with longer career in the role. Role ambiguity did not retain an independent association once professional autonomy and the work environment were taken into account (*β* = −0.11, *p* = 0.135), nor did the remaining covariates. Together the nine variables explained 55.6% of the variance in job satisfaction (*F*(9, 120) = 18.92, *p* < 0.001) (Table 5).

Residuals were consistent with normality (Shapiro–Wilk *W* = 0.990, *p* = 0.498; skewness −0.22, kurtosis 0.64) and with constant variance (Breusch–Pagan Lagrange multiplier = 4.85, *p* = 0.847), and the Ramsey RESET test gave no evidence of functional-form misspecification (*F* = 1.65, *p* = 0.197). The maximum Cook’s distance was 0.094 against a conventional threshold of 1.0, the maximum leverage was 0.250, and a single case had an externally studentized residual beyond ±3; excluding it did not alter any conclusion. Estimates were stable under heteroscedasticity-consistent (HC3) standard errors and under percentile bootstrap confidence intervals from 5000 resamples, and no coefficient changed significance status under either procedure (Table A1).

Harman’s single-factor test was applied to all 99 items of the four instruments. Twenty-two factors had eigenvalues greater than 1 and the first accounted for 31.1% of the total variance, below the 50% threshold conventionally taken to indicate that a single method factor dominates the covariance among measures.

When each main variable was entered alone and the other two were then added in turn, all three coefficients fell. Professional autonomy fell from *β* = 0.67 to 0.39 and the work environment from *β* = 0.67 to 0.37, both remaining significant at every step (all *p* < 0.001). Role ambiguity, entered alone, accounted for 25.6% of the variance in job satisfaction (*β* = −0.51, *p* < 0.001); this fell to *β* = −0.25 (*p* < 0.001) with professional autonomy in the model, to *β* = −0.18 (*p* = 0.025) with the work environment, and to *β* = −0.11 (*p* = 0.157) with both, at which point it was no longer significant. Professional autonomy and the work environment together explained 36.1% of the variance in role ambiguity. The unique contribution of role ambiguity to job satisfaction in the full model was Δ*R*^2^ = 0.008, compared with 0.105 for professional autonomy and 0.067 for the work environment. Details are given in Table A2.

## 4. Discussion

This study examined professional autonomy, work environment, role ambiguity, and job satisfaction among nurses in physician-support roles and the variables associated with job satisfaction. Professional autonomy and the work environment were independently associated with job satisfaction, and the model including the six general characteristics accounted for 55.6% of the variance; role ambiguity did not retain an independent association once the other two constructs were taken into account.

Job satisfaction averaged 3.56 out of 5, above the midpoint but below the 3.69 to 4.12 reported for clinical nurses with the same instrument [36,37]. Within the scale, job security and reward scored highest (3.95) while organizational recognition and professional achievement scored lowest (3.17), a difference of 0.78 on the same five-point metric. What these nurses rated lowest was therefore not material conditions but organizational recognition of the expertise those conditions are meant to reward, a pattern consistent with accumulated expertise in the role going unrecognized and with limited opportunities for promotion and long-term career development [6]. Retaining experienced nurses may therefore require career pathways that recognize accumulated expertise, alongside the legal foundation for autonomy and the organizational recognition discussed above.

Professional autonomy showed the strongest independent association with job satisfaction. Correlations of this kind have been reported in this workforce [28], but the two studies that entered autonomy alongside other work-related variables did not find it independently associated with job satisfaction [18,19]. Both measured autonomy with the Schutzenhofer Professional Nurse Autonomy Scale, which asks how often specific clinical actions are carried out independently of physician direction [17], whereas the present study used the Dempster Practice Behaviors Scale, whose empowerment subscale addresses institutionally conferred authority [32]. The variables entered alongside autonomy also differed: nurse–physician collaboration in one study and job crafting in the other, both of which overlap conceptually with autonomy. The subscale pattern of the present measure helps explain why autonomy emerged as the strongest independent correlate here: actualization scored highest and empowerment lowest, with the lowest ratings of all on items concerning legal grounds and institutionally conferred authority. These nurses therefore exercise professional judgment and accept responsibility in practice while perceiving no clear legal basis for doing so [1]. The contrast with the United States is instructive: the American physician assistant system secures legal legitimacy through formal education, national certification, licensure, and continuing education [5], whereas no comparable framework existed in Korea at the time of data collection. The enforcement rule in force since 11 August 2026 has addressed part of that gap, setting eligibility at three years of clinical experience and completion of a designated curriculum and defining physician-support work as a list of permitted acts [4]. The present findings therefore describe the situation immediately before that framework took effect and provide a baseline against which change in perceived autonomy can be assessed.

The work environment showed the second strongest independent association, consistent with previous research in this workforce [16] and with a six-state study of 1244 nurse practitioners in which relations with administrators and relations with physicians, measured on the same instrument, were associated with job satisfaction and intent to leave [38]. The mean of 2.61 was similar to, or slightly above, the 2.45 to 2.48 reported with the same instrument [16,39]. Among its subscales, relationships with administrators scored lowest: these nurses perceive that they are not treated as fairly as physicians by managers and that organizational information is not adequately shared with them. This was also the lowest scoring subscale in the six-state study, where it showed by far the strongest association with job satisfaction [38]. This may reflect a dual structure in which most are affiliated with the department of nursing while their work is directed and evaluated by physicians [40], leaving both administrative and nursing managers without a clear view of the role. A role that sits between two professions is particularly liable to fall outside established lines of supervision and recognition. A national survey of this workforce identified the mismatch between the department of affiliation and the department issuing work instructions as a recurring difficulty, and rated the establishment of a liaison structure with the department of nursing as the least well developed of the measures needed to define the role [1]. Relationships with physicians scored highest, which is plausible given daily collaboration and consistent with the subscale ordering reported in Korean studies using the same instrument [29,39], but items on whether physicians trust their decisions and recognize their value scored low. Models of nurse practitioner–physician co-management treat effective communication and mutual respect and trust as separate elements [41], and the present pattern suggests that collaboration does not extend to recognition of expertise. These descriptive patterns suggest that aligning operational systems with the role and to strengthen relationships between administrators and these nurses are the aspects of the work environment most in need of attention, although the present design cannot establish what changing them would achieve.

Role ambiguity correlated negatively with job satisfaction, consistent with studies of physician assistants abroad [42], and was higher in this workforce than in nurses working in comprehensive nursing care service wards [23,43]. Within the scale, perceptions of the boundaries of their own authority were least clear, whereas performance was relatively stable in practical domains such as work planning and time allocation; these nurses thus exercise discretion while perceiving the limits of their responsibility as ambiguous [12]. Role ambiguity was associated with job satisfaction at the bivariate level (*r* = −0.51, *p* < 0.001) but did not retain an independent association once professional autonomy and the work environment were taken into account (*β* = −0.11, *p* = 0.135; *B* = −0.07, 95% CI −0.17 to 0.02). This is a statement about how variance is partitioned among three interrelated constructs in these data, and not a statement about what would follow from measures that reduce role ambiguity. The confidence interval remains compatible with a small negative association, and the study was powered for the overall regression model rather than for the unique contribution of any single construct: with 130 participants and nine predictors, the smallest unique contribution detectable with 80% power was Δ*R*^2^ ≈ 0.025, against an observed value of 0.008. That small unique contribution reflects variance shared with the other two constructs: professional autonomy and the work environment together account for more than a third of the variance in role ambiguity (Table A2). Whether role ambiguity is better understood as arising from the work environment, or as a determinant standing alongside it, cannot be resolved with constructs measured at a single time point, and we offer that question as a hypothesis for longitudinal research rather than as a finding of the present study. The enforcement rule now requires every hospital performing physician-support work to establish an operating committee and prepare a job description through it [4], so the instrument this study would otherwise have recommended is already mandated, and what remains is to evaluate whether it changes what these nurses experience.

Job satisfaction was lower among nurses with longer service in the physician-support role, an association that was absent in the bivariate comparison and emerged only with professional autonomy and the work environment held constant. Two mechanisms are plausible and cannot be distinguished with these data. The first is cumulative occupational strain: burnout in this workforce has been reported to rise with sustained exposure to role conflict and to indistinct role boundaries [24], and the physician associate literature reports a comparable accumulation of strain over time in the role [42]. Where expertise accumulates without a corresponding route into management, organizational recognition—the lowest scoring job satisfaction subscale in this sample—is the component of satisfaction most exposed to erosion. The second mechanism is selective attrition: those who remain longest in the role are a survivor population, and job stress in this workforce has been shown to predict turnover intention through job satisfaction [13], so that at any given tenure the least satisfied are the most likely already to have left. Distinguishing a genuine decline with time in the role from this composition effect requires longitudinal data, and the coefficient is therefore best read as descriptive of the sample rather than as evidence about the trajectory of individuals.

### 4.1. Implications for Practice

The associations reported here are conditional and cross-sectional, and they do not identify what would follow from changing any of the constructs measured. The considerations below are therefore offered as hypotheses, informed partly by the descriptive pattern of subscale scores in this sample and partly by prior research, rather than as effects estimated by this study. First, professional autonomy may require a legal foundation that matches the judgment these nurses already exercise. The enforcement rule has established eligibility criteria and a list of permitted acts, but the low ratings for institutionally conferred authority recorded here suggest that formal permission and perceived authority are not the same thing; one candidate measure would be for hospitals to make explicit, within the scope the rule allows, what these nurses may decide without referral. Second, the work environment might be addressed through the relationship with administrators, which scored lowest of all subscales. Two measures that would follow from this reading, neither of which has been evaluated, would be to organize these nurses into dedicated teams led by a team leader appointed from among their own number, a post that does not at present exist within the physician-support role, and to include them in the channels through which organizational information is shared. Third, because organizational recognition and professional achievement scored lowest of all job satisfaction subscales, career pathways that recognize accumulated expertise may be worth evaluating as a means of retaining experienced nurses, and a team leader post within the physician-support role itself would provide the route into management that this workforce currently lacks. None of these measures has been evaluated in this workforce, and the present design cannot estimate what any of them would achieve; each is a candidate for prospective evaluation rather than a conclusion of this analysis.

Future research should examine whether the associations reported here persist under the enforcement rule. Repeating these measurements after implementation would show whether the statutory requirements for job descriptions, operating committees, and preparatory education deliver the role clarity and professional autonomy this study found wanting. Because the cross-sectional design of the present study cannot establish the ordering among the three variables, longitudinal designs in which the constructs are measured at separate time points are needed before any pathway among them can be tested.

### 4.2. Limitations

This study has several limitations of design and sampling. It used a convenience sample drawn from two university hospitals of approximately 900 beds in a single metropolitan area. The two are among the three hospitals of that size in the city, so the sample reflects that institutional tier reasonably well, but the findings should not be read at national level without further study. Data were collected in July–August 2025, after the Nursing Act took effect on 21 June 2025 but before the enforcement rule on nurse-delivered physician-support work came into force on 11 August 2026, so the findings should be generalized with caution. The cross-sectional design precludes causal inference among the study variables, and because all variables were measured at one time point the ordering among them cannot be established. All measures were also self-reported and collected at a single time point, so common method bias cannot be excluded.

Five features of the estimates themselves warrant caution in interpretation. First, the three main variables are interrelated, so each regression coefficient represents an association conditional on the other two rather than a total association; if an unfavorable work environment contributes to role ambiguity, the coefficient reported for the work environment understates its overall association with job satisfaction. Second, career in the physician-support role was entered as a covariate rather than as a variable under study, so its own confounders were not modeled; its association with job satisfaction was absent in the bivariate comparison and emerged only with the other variables held constant, and cross-sectional data cannot separate a decline with time in the role from selective attrition of those who were less satisfied. Third, sex was likewise entered as a covariate and the study was not designed to examine sex differences; with 35 male participants, any sex-stratified analysis would have been underpowered, so whether the associations reported here hold equally for men and women remains to be established. Fourth, the sample size was determined for the overall regression model and not for the unique contribution of any individual construct. With 130 participants and nine predictors, the smallest unique contribution detectable with 80% power at *α* = 0.05 was *f*^2^ = 0.061 (Δ*R*^2^ ≈ 0.025); the unique contribution of role ambiguity observed here was Δ*R*^2^ = 0.008, for which post hoc power was 0.34. The non-significant adjusted coefficient for role ambiguity should therefore be read as an imprecise estimate rather than as evidence that no independent association exists. No formal equivalence test was conducted, because the literature provides no established margin below which such an association could be judged negligible. Fifth, age, total clinical career and career in the physician-support role were grouped into categories for the descriptive and bivariate analyses, which loses information and makes those comparisons dependent on the chosen cut-points; the regression model entered career in the physician-support role as a continuous variable in years and entered neither age nor total clinical career, so the estimates reported here do not depend on those categories. Further replication across a range of time points and institutions is recommended, so that job satisfaction in this workforce can be examined on a continuing basis.

Participants were recruited from two university hospitals, and professional autonomy, the work environment, and job satisfaction are all shaped by institutional characteristics, so differences between the two sites could confound the individual-level associations reported here; the association of the work environment with job satisfaction is the most exposed to this, since the work environment is by definition an organizational construct. A hospital site identifier is not available, however, because a two-level indicator combined with the characteristics collected would have made individual respondents identifiable (Section 2.5), so site could not be entered as a fixed effect and site-level clustering could not be modeled. The composition of the analytic sample can nonetheless be bounded: 100 questionnaires were provided at one hospital and 43 at the other and all were returned; the 130 analyzed comprise between 87 and 100 respondents from the larger site and between 30 and 43 from the smaller. Both hospitals are university hospitals of comparable size operating under the same national regulatory framework for physician-support nursing, so the contrast between them is limited, and in a quantitative bias analysis using that composition and a range of assumed between-site differences in the work environment score (a hypothetical site indicator simulated 5000 times) professional autonomy remained significantly associated with job satisfaction in every construction and the work environment in 99.4% of constructions, including 98.9% of those in which the induced difference exceeded one standard deviation. Unmeasured site clustering is therefore unlikely to account for the principal findings, although it cannot be excluded as a contributor to the less precise estimates.

## 5. Conclusions

This study described levels of professional autonomy, work environment, role ambiguity, and job satisfaction among nurses in physician-support roles and examined which of them were independently associated with job satisfaction. Job satisfaction was above the midpoint. Professional autonomy and the work environment were positively correlated with job satisfaction and role ambiguity was negatively correlated with it. In the mutually adjusted model, professional autonomy showed the strongest independent association with job satisfaction, followed by the work environment, whereas role ambiguity did not retain an independent association once the other two constructs were taken into account. These results direct attention to the legal and institutional conditions that support professional autonomy and a favorable work environment. Role ambiguity was associated with job satisfaction when considered alone, and its variance overlaps substantially with that of the other two constructs, which together account for 36.1% of the variance in role ambiguity. The results therefore do not indicate that role clarity is unimportant; they indicate that in this setting role ambiguity carried little information about job satisfaction beyond what professional autonomy and the work environment already carried, and they suggest that clarifying the role is unlikely on its own to be sufficient where professional autonomy and the organizational conditions supporting it remain absent. Standardized job descriptions specifying scope of practice, responsibility, and authority are now required of every hospital performing physician-support work. Whether measures directed at any of the three constructs would change job satisfaction is a question this design cannot answer, and what remains is to evaluate whether these requirements, applied alongside measures that secure professional autonomy and improve the work environment, translate into higher job satisfaction in practice.

The study was conducted after the Nursing Act had taken effect but before the enforcement rule specifying scope of practice did. It therefore offers a reference point against which the role can be compared as the rule takes hold.

## Figures and Tables

**Table 1:** General characteristics of participants (*N* = 130).

Variable	Category	*n* (%)	Mean ± SD
Age (years)	≤30	51 (39.2)	32.73 ± 4.73
	>30 to ≤35	44 (33.8)	
	>35	35 (26.9)	
Sex	Male	35 (26.9)	
	Female	95 (73.1)	
Education level	≤Bachelor’s degree	122 (93.8)	
	≥Master’s degree	8 (6.2)	
Marital status	Unmarried	66 (50.8)	
	Married	64 (49.2)	
Children	Yes	43 (33.1)	
	No	87 (66.9)	
Position	Staff or charge nurse	102 (78.5)	
	≥ Senior charge nurse	28 (21.5)	
Total clinical career (years)	≤7	47 (36.2)	9.75 ± 4.71
	>7 to ≤11	41 (31.5)	
	>11	42 (32.3)	
Career in physician-support role (years)	≤3	56 (43.1)	5.28 ± 4.03
	>3 to ≤7	39 (30.0)	
	>7	35 (26.9)	
Current department	Medical or intensive care unit	47 (36.2)	
	Surgical unit	83 (63.8)	
Department of affiliation	Medical department	3 (2.3)	
	Nursing department	127 (97.7)	
Working type	Fixed daytime	119 (91.5)	
	Rotating shifts or other	11 (8.5)	
Motivation for entering the role	Professional reasons ^a^	50 (38.5)	
	To avoid rotating shifts	50 (38.5)	
	Assigned by the hospital or other	30 (23.1)	
Annual salary (10,000 KRW)	<4500	27 (20.8)	
	4500 to <5500	54 (41.5)	
	≥5500	49 (37.7)	
Pre-placement job training	Yes	14 (10.8)	10.29 ± 6.82 ^b^
	No	116 (89.2)	
Post-placement job training	Yes	64 (49.2)	8.72 ± 9.81 ^c^
	No	66 (50.8)	
Written work manual	Available	83 (63.8)	
	Not available	47 (36.2)	

SD, standard deviation; KRW, Korean won. Percentages are calculated on *N* = 130 and may not sum to 100 because of rounding. ^a^ Aptitude and interest, more specialized work than a general nurse, and opportunity for self-development combined. ^b^ Hours among the 14 participants who received pre-placement training. ^c^ Hours among the 64 participants who received post-placement training.

**Table 2:** Levels of study variables (*N* = 130).

Variable	Range	Item Mean ± SD	Observed Range
Professional autonomy	1–5	3.40 ± 0.65	1.73–5.00
Readiness	1–5	3.35 ± 0.75	1.64–5.00
Empowerment	1–5	3.05 ± 0.68	1.43–5.00
Actualization	1–5	3.71 ± 0.65	2.00–5.00
Valuation	1–5	3.43 ± 0.99	1.00–5.00
Work environment	1–4	2.61 ± 0.49	1.34–3.86
Professional visibility	1–4	2.62 ± 0.61	1.00–4.00
Relationships with administrators	1–4	2.22 ± 0.64	1.00–4.00
Relationships with physicians	1–4	2.94 ± 0.64	1.00–4.00
Independent practice and support	1–4	2.74 ± 0.49	1.11–4.00
Role ambiguity	1–5	2.98 ± 0.87	1.00–5.00
Job satisfaction	1–5	3.56 ± 0.56	2.09–5.00
Organizational recognition and professional achievement	1–5	3.17 ± 0.76	1.11–5.00
Personal maturity through work	1–5	3.54 ± 0.72	1.67–5.00
Relationships based on respect and recognition	1–5	3.68 ± 0.54	2.13–5.00
Fulfillment of responsibility as a professional nurse	1–5	3.92 ± 0.62	2.25–5.00
Exercise of professional competence	1–5	3.59 ± 0.61	2.00–5.00
Job security and reward	1–5	3.95 ± 0.70	2.33–5.00

SD, standard deviation. Subscales are indented under their parent scale. Items 8, 13, 17, and 28 of the Dempster Practice Behaviors Scale were reverse-scored before analysis; all four belong to the empowerment subscale.

**Table 3:** Differences in job satisfaction by general characteristics (*N* = 130).

Variable	Category	Item Mean ± SD	*t* or *F* (*p*)
Age (years)	≤30	3.62 ± 0.57	0.93 (0.396)
	>30 to ≤35	3.47 ± 0.51	
	>35	3.58 ± 0.60	
Sex	Male	3.61 ± 0.55	0.67 (0.503)
	Female	3.54 ± 0.56	
Education level	≤Bachelor’s degree	3.55 ± 0.53	−0.32 (0.755)
	≥Master’s degree	3.67 ± 0.98	
Marital status	Unmarried	3.56 ± 0.51	0.08 (0.936)
	Married	3.56 ± 0.61	
Children	Yes	3.48 ± 0.58	−1.15 (0.254)
	No	3.60 ± 0.55	
Position	Staff or charge nurse	3.57 ± 0.56	0.27 (0.784)
	≥Senior charge nurse	3.53 ± 0.56	
Total clinical career (years)	≤7	3.64 ± 0.55	1.28 (0.282)
	>7 to ≤11	3.45 ± 0.47	
	>11	3.57 ± 0.64	
Career in physician-support role (years)	≤3	3.64 ± 0.52	0.91 (0.404)
	>3 to ≤7	3.49 ± 0.58	
	>7	3.52 ± 0.59	
Current department	Medical or intensive care unit	3.48 ± 0.50	−1.19 (0.236)
	Surgical unit	3.60 ± 0.59	
Department of affiliation	Medical department	3.72 ± 0.51	0.49 (0.625)
	Nursing department	3.56 ± 0.56	
Working type	Fixed daytime	3.56 ± 0.56	0.14 (0.886)
	Rotating shifts or other	3.54 ± 0.52	
Motivation for entering the role	Professional reasons	3.69 ± 0.49	2.49 (0.087)
	To avoid rotating shifts	3.50 ± 0.58	
	Assigned by the hospital or other	3.43 ± 0.59	
Annual salary (10,000 KRW)	<4500	3.39 ± 0.49	1.64 (0.199)
	4500 to <5500	3.61 ± 0.57	
	≥5500	3.60 ± 0.57	
Pre-placement job training	Yes	3.48 ± 0.69	−0.53 (0.594)
	No	3.57 ± 0.54	
Post-placement job training	Yes	3.66 ± 0.54	2.13 (0.035)
	No	3.46 ± 0.57	
Written work manual	Available	3.59 ± 0.55	0.73 (0.465)
	Not available	3.51 ± 0.57	

SD, standard deviation; KRW, Korean won. Analyzed with independent-samples *t*-tests and one-way analysis of variance. For education level, Levene’s test indicated unequal variances (*F* = 14.94, *p* < 0.001) and the *t* statistic for unequal variances is reported (*df* = 7.27).

**Table 4:** Correlations among study variables (*N* = 130).

Variable	Professional Autonomy	Work Environment	Role Ambiguity
	*r* (*p*)		
Work environment	0.60 (<0.001)		
Role ambiguity	−0.48 (<0.001)	−0.59 (<0.001)	
Job satisfaction	0.67 (<0.001)	0.67 (<0.001)	−0.51 (<0.001)

*r*, Pearson correlation coefficient.

**Table 5:** Variables associated with job satisfaction (*N* = 130).

Variable	B	SE	95% CI	*β*	*t*	*p*	VIF
(Constant)	1.55	0.37	0.82, 2.27		4.24	<0.001	
Professional autonomy	0.36	0.07	0.23, 0.49	0.42	5.51	<0.001	1.67
Work environment	0.42	0.09	0.23, 0.60	0.37	4.42	<0.001	1.99
Role ambiguity	−0.07	0.05	−0.17, 0.02	−0.11	−1.51	0.135	1.69
Sex (female)	0.06	0.08	−0.10, 0.21	0.05	0.74	0.463	1.14
Education (≥master’s degree)	0.09	0.14	−0.20, 0.37	0.04	0.62	0.537	1.11
Position (senior charge nurse)	0.06	0.09	−0.11, 0.24	0.04	0.69	0.491	1.21
Career in the role (years)	−0.02	0.01	−0.04, −0.003	−0.15	−2.29	0.024	1.29
Post-placement training (yes)	−0.06	0.07	−0.20, 0.09	−0.05	−0.77	0.440	1.20
Work manual (available)	−0.01	0.07	−0.14, 0.13	−0.01	−0.11	0.909	1.02
Model fit: *R*^2^ = 0.587; adjusted *R*^2^ = 0.556; *F*(9, 120) = 18.92, *p* < 0.001.							

B, unstandardized coefficient; SE, standard error; CI, confidence interval; *β*, standardized coefficient; VIF, variance inflation factor. The dependent variable is the job satisfaction item mean. The first three variables are those under study; the remaining six were specified in advance as covariates. All nine were entered simultaneously. Reference categories: male; bachelor’s degree or lower; staff or charge nurse; no post-placement training; no work manual.

## Data Availability

The datasets generated and analyzed during the current study are not publicly available because participants did not consent to public release of their individual data, but are available from the corresponding author on reasonable request.

## Abbreviations

The following abbreviations are used in this manuscript:

DPBS	Dempster Practice Behaviors Scale
HC3	Heteroscedasticity-consistent covariance estimator
NP-PCOCQ	Nurse Practitioner Primary Care Organizational Climate Questionnaire
PA	Physician assistant
SD	Standard deviation
STROBE	Strengthening the Reporting of Observational Studies in Epidemiology
VIF	Variance inflation factor

## Appendix A

**Table A1:** Sensitivity analyses for the model of variables associated with job satisfaction (*N* = 130).

Variable	B (SE)	OLS 95% CI	HC3 SE	HC3 95% CI	Bootstrap 95% CI
Professional autonomy	0.362 (0.066)	0.232, 0.492	0.080	0.204, 0.520	0.211, 0.502
Work environment	0.417 (0.094)	0.230, 0.603	0.122	0.176, 0.658	0.201, 0.654
Role ambiguity	−0.074 (0.049)	−0.171, 0.023	0.056	−0.185, 0.037	−0.176, 0.032
Sex (female)	0.058 (0.079)	−0.098, 0.214	0.081	−0.102, 0.218	−0.095, 0.209
Education (≥master’s degree)	0.089 (0.144)	−0.195, 0.373	0.157	−0.222, 0.400	−0.228, 0.375
Position (senior charge nurse)	0.061 (0.088)	−0.113, 0.234	0.094	−0.125, 0.247	−0.115, 0.237
Career in the role (years)	−0.021 (0.009)	−0.039, −0.003	0.009	−0.039, −0.003	−0.039, −0.003
Post-placement training (yes)	−0.055 (0.072)	−0.197, 0.086	0.077	−0.209, 0.098	−0.207, 0.084
Work manual (available)	−0.008 (0.069)	−0.144, 0.128	0.072	−0.151, 0.135	−0.140, 0.130

B, unstandardized coefficient; SE, standard error; OLS, ordinary least squares; CI, confidence interval; HC3, heteroscedasticity-consistent covariance estimator. The dependent variable is the job satisfaction item mean; all nine variables were entered simultaneously and the constant is omitted. Bootstrap intervals are percentile intervals from 5000 resamples. Model fit: *R*^2^ = 0.587; adjusted *R*^2^ = 0.556; *F*(9, 120) = 18.92, *p* < 0.001.

**Table A2:** The change in each coefficient as the three main variables alone are entered in turn (*N* = 130).

Variables Entered	Adjusted *R*^2^	B (SE)	*β*	*p*
Professional autonomy alone	0.438	0.576 (0.057)	0.665	<0.001
+work environment	0.548	0.358 (0.064)	0.413	<0.001
+role ambiguity	0.483	0.472 (0.063)	0.546	<0.001
+both	0.551	0.340 (0.065)	0.392	<0.001
Work environment alone	0.441	0.760 (0.075)	0.668	<0.001
+professional autonomy	0.548	0.477 (0.084)	0.419	<0.001
+role ambiguity	0.459	0.638 (0.091)	0.561	<0.001
+both	0.551	0.420 (0.093)	0.369	<0.001
Role ambiguity alone	0.256	−0.329 (0.049)	−0.512	<0.001
+professional autonomy	0.483	−0.160 (0.046)	−0.249	<0.001
+work environment	0.459	−0.117 (0.051)	−0.182	0.025
+both	0.551	−0.068 (0.048)	−0.106	0.157

B, unstandardized coefficient; SE, standard error; *β*, standardized coefficient. Each row is a separate regression with job satisfaction as the dependent variable; the coefficient shown is that of the first variable in each panel and adjusted *R*^2^ refers to the model as a whole. The six covariates of Table 5 were not entered so that the change in each coefficient reflects only the overlap among the three variables under study; these reduced models describe that overlap and are not comparable with the adjusted estimates of Table 5.

### Quantitative Bias Analysis for Unmeasured Hospital Site

The hospital at which each respondent worked was not recorded, so the following construction was used to ask how large an unmeasured site difference would have to be before it overturned the findings. In each of 5000 constructions, between 30 and 43 of the 130 respondents were assigned to the smaller hospital, the bounds implied by the numbers of questionnaires distributed at the two sites (Section 2.3). Assignment was made by ranking respondents on θ·z(work environment) + λ·z(job satisfaction) plus independent standard Gumbel noise and taking the highest, which is sampling without replacement with probability proportional to exp(θ·z + λ·z); θ and λ were each drawn from a uniform distribution on (−3, +3), so that in a given construction the two simulated sites might differ on either variable by anything from nothing to more than one and a half standard deviations. No observed value was altered. The primary model was then refitted with the indicator entered as a tenth predictor, and the standardized difference in the work environment between the two simulated sites was recorded alongside the significance of professional autonomy and the work environment.
